# Dynamic, continuous multitasking training leads to task-specific improvements but does not transfer across action selection tasks

**DOI:** 10.1038/s41539-017-0015-4

**Published:** 2017-12-04

**Authors:** Angela D. Bender, Hannah L. Filmer, Claire K. Naughtin, Paul E. Dux

**Affiliations:** School of Psychology, The University of Queensland, Queensland, Australia

## Abstract

The ability to perform multiple tasks concurrently is an ever-increasing requirement in our information-rich world. Despite this, multitasking typically compromises performance due to the processing limitations associated with cognitive control and decision-making. While intensive dual-task training is known to improve multitasking performance, only limited evidence suggests that training-related performance benefits can transfer to untrained tasks that share overlapping processes. In the real world, however, coordinating and selecting several responses within close temporal proximity will often occur in high-interference environments. Over the last decade, there have been notable reports that training on video action games that require dynamic multitasking in a demanding environment can lead to transfer effects on aspects of cognition such as attention and working memory. Here, we asked whether continuous and dynamic multitasking training extends benefits to tasks that are theoretically related to the trained tasks. To examine this issue, we asked a group of participants to train on a combined continuous visuomotor tracking task and a perceptual discrimination task for six sessions, while an active control group practiced the component tasks in isolation. A battery of tests measuring response selection, response inhibition, and spatial attention was administered before and immediately after training to investigate transfer. Multitasking training resulted in substantial, task-specific gains in dual-task ability, but there was no evidence that these benefits generalized to other action control tasks. The findings suggest that training on a combined visuomotor tracking and discrimination task results in task-specific benefits but provides no additional value for untrained action selection tasks.

## Cognition: multitasking training boosts task-specific skills only

Training on a dynamic multitasking game leads to task-specific improvements but this performance gain does not transfer to novel tasks. Bender and colleagues from the University of Queensland, Australia, had one group of human participants train on a combined visuomotor tracking and shape discrimination task over several days, while another group trained on the two tasks in isolation (active control group). Following training, multitasking performance selectively improved for the multitasking group and not for the active control group. Critically, this training-related benefit did not generalize to a wide range of cognitive tasks that are theoretically linked to the current dual-task paradigm, indicating that repeated exposure to two concurrent tasks induces the learning of task-specific skills so that coordination between two specific tasks can be implemented more efficiently.

## Introduction

The modern, information-rich world demands that we often have to undertake multiple tasks concurrently. Despite this, the effective selection of task-relevant responses (i.e., decision-making/response selection) and the suppression of task-irrelevant information/responses (i.e., response inhibition) are often significantly compromised when humans attempt to execute multiple cognitive operations simultaneously. Multitasking ability can be assessed in a wide range of action selection paradigms that place strong demands on central information processing resources. For instance, traditional laboratory-based measures of multitasking ability often include dual-task paradigms that require participants to perform two simple choice reaction time (RT) tasks simultaneously, relative to by themselves,^1–4^ whereas in the commonly used psychological refractory period (PRP) method^5,6^ participants perform speeded responses to two tasks/stimuli that occur in relatively close or far temporal proximity. While conditions differ across paradigms, a consistent finding is the observed dual-task cost—performance impairments in one or both tasks, as indexed by a decrease in accuracy and/or increase in RT when two tasks need to be performed simultaneously or close in time, relative to when the two tasks are performed far apart or in isolation.

Fortunately, evidence suggests that dual-task costs can be reduced with practice/training, with participants consistently displaying experience-related improvements on the task itself.^3,7–11^ Neuroimaging studies investigating the neural underpinnings associated with reduced multitasking costs have found that dual-task training decreases cortical activity in sub-regions of the dorsolateral prefrontal cortex, posterior lateral prefrontal cortex, basal ganglia, and parietal cortex,^2,12,13^ a network of areas that is frequently recruited in a wide range of tasks that require the executive control of action.^14–17^ Although these studies offer intriguing insights into the potentials of dual-task training, the exact mechanisms that contribute to training-related behavioral and neural adaptation effects are currently not fully understood. Several explanations for these effects have been proposed: (1) capacity-limited response selection processes are shortened in the single component tasks;^2,18,19^ (2) the repeated exposure to consistent stimulus-response mappings in each component task leads to the development of memory traces that enable the direct associations between stimuli and responses;^18,20–22^ and (3) dual-task performance leads to the acquisition of improved attentional control skills in coordinating two independent tasks concurrently.^8,10,23^ Thus, dual-task performance is improved by reducing the conflict for limited central resources. The proposed mechanisms are in line with a recent large-scale neuroimaging study by Garner and Dux,^12^ which suggests that training increases the separation of the neural representations of the constituent tasks, indicating that more fine-tuned task representations contribute to a reduction in dual-task interference, which in turn may facilitate the coordination of two practiced tasks.

While task-specific dual-task interference can be reduced with training, the extent to which dual-task training generalizes to other non-trained multitasking measures or secondary measures of cognitive processes that are closely linked to multitasking ability is still hotly debated. It has been hypothesized generally that the probability of transfer from one measure to another is increased when tasks draw on the same cognitive processes and overlapping neural substrates as the trained task.^24–26^ Moreover, training-related enhancements to other tasks seem to predominantly occur if the untrained tasks share a strong similarity with the trained task in terms of response modality (e.g., responding via keyboard press), input modality (e.g., both tasks employ visual stimuli), timings with the trained task,^27^ or overlap in terms of common or abstract rules.^28^ In contrast, training gains are disrupted when the practiced stimuli are presented in another modality (e.g., visual to auditory^28^). These findings indicate that trained responses are not fully automatic and that the observed modality transfer effects may be due to an improved capacity-limited central response selection mechanism that integrates modality-specific information to a response.

So far however, the majority of traditional laboratory multitasking training studies show little evidence for significant transfer after dual-task practice,^8,18,29–31^ with only a few studies reporting positive, but often limited, training transfer gains to other multitasking situations.^8,27^ For example, a recent set of studies^8,10,32^ found that training-related enhancements of multitasking skills transferred to novel two choice RT tasks only when one of the component tasks was changed from practice. However, no transfer to other tasks was observed when changes occurred in both component tasks, indicating that training-related task coordination skills seem to be task-specific and non-transferable.

While the observations described above suggest that training-related transfer effects from traditional multitasking tests are possible but rather limited, larger transfer gains have been shown when assessing the impact of video action game training on executive control. Action gaming typically requires the performance of several actions simultaneously, such as the continuous tracking of a moving target while monitoring and responding to game-related stimuli to achieve the required goals and sub-goals.^33^ The continuous requirement in action games to constantly monitor and coordinate several tasks within close temporal proximity heavily tax perception, attention and capacity-limited response selection processes. Indeed, contrasting performance gains of video action and non-action gamers, such video action gaming interventions have been shown in a number of studies to not only lead to training-related performance improvements on the trained task but have also generalized to other aspects of central executive control such as multitasking and task switching^34–36^ (but see ref. 37), attention,^38–40^ and working memory.^33,41^

However, despite the potential benefits of action video games, action gaming may not be the right intervention for everyone, given its often violent themes. More recently, Anguera and colleagues^42^ introduced a hybrid approach that incorporated some of the characteristics thought to contribute to positive transfer gains after video action game training (e.g., a stimulating and high-interference environment that heavily taxes central processing demands, feedback and timely rewards) in a 3D video game training paradigm designed to assess and improve cognitive control skills in older adults. In this prominent study,^42^ participants had to keep a moving car in the center of a winding road while simultaneously responding to a speeded shape discrimination task as quickly and accurately as possible (multitasking condition) or perform the component tasks in isolation (single-task condition). The results showed that multitasking ability improved after the two tasks had been trained in combination (multitasking group) relative to when the two tasks had been trained in isolation (single-task group) or not at all (no contact control group). Crucially, while training-related improvements in multitasking did not generalize to a novel dual-task paradigm, analyses of transfer effects revealed significant post-training gains on measures of working memory and sustained attention after video game training in multitasking training mode—two cognitive operations that are thought to reflect central executive control capabilities.^43^

Taken together, an overview of the cognitive training literature indicates that the engagement in tasks that require online, dynamic decision-making within a demanding environment leads to the best chance of positive transfer to other tasks that recruit similar neural networks and are theoretically related to the trained tasks. However, there are a number of key factors worth considering before one can definitively conclude that training transfer is possible following a cognitive training regime. As several recent reviews of cognitive training discuss,^44–46^ a gold standard cognitive intervention should (1) employ outcome measures with a clear targeted theoretical construct; (2) minimize potential placebo effects and potential treatment confounds by including an active control group that ensures equal task engagement and enjoyment; (3) randomly allocate participants to treatment or control to validate trained and transfer gains; and (4) transfer tasks should be restricted to tasks that are related to the trained construct.

While the previous dual-task training literature has regularly shown that training on traditional multitasking paradigms leads to task-specific reductions in dual-task costs,^3,7–11^ it is still an open question regarding whether or not multitasking costs can also be attenuated in an experimental task that requires rapid decision-making in a high-interference environment, and if so whether these benefits extend to tasks that are theoretically related to the trained tasks.

Motivated by the video game training literature and guided by the latest cognitive training principles, we created a dynamic paradigm, which employs a continuous, dynamic visuomotor tracking task in conjunction with a perceptual discrimination task to tax perception, response selection and visuospatial processes, as such a dynamic task is more reflective of complex real-world situations (e.g., driving a car and concurrently monitoring the environment for task-relevant cues). Evaluating the possible transfer effects of a dynamic multitasking training paradigm to other cognitive domains is important because it provides a broader perspective on how different experimental designs produce different effects, which in turn advances our knowledge about the underlying learning mechanisms that give rise to positive transfer effects. Thus, to determine whether training-induced benefits extend to other action control tasks, participants completed a battery of psychological tasks at pre-training and post-training. In a previous factor-analytic study,^47^ we showed that the construct of response selection could be measured via tasks such as the PRP, single and dual response selection, the Stroop and to a lesser degree the Attentional Blink (AB). Hence, to examine the transferability of any training-induced multitasking benefits to other response selection measures, participants completed the PRP, a six alternative forced choice (AFC) single response selection task, the Stroop task and the AB. Secondary processes of cognitive operations linked with response selection, including tests of response inhibition (Go-Nogo task) and measures of selective attention (Flanker task) were also included.

## Results

### Multitasking cost

To investigate the effects of training on multitasking performance, we first explored performance before training on the sample as a whole (Table 1 and Supplementary Table 1). The overall mean accuracy for the single discrimination task and single tracking task was 83% and 80%, respectively, demonstrating that the adaptive thresholding procedure successfully determined a detection and tracking level of ~80% accuracy on each component task, with no significant baseline differences between the two training groups on either discrimination accuracy (*t*(37) = −1.86, *p* = .07) or tracking accuracy (*t*(37) = 0.84, *p* = .40). At pre-training, simultaneous performance of the shape discrimination task and the visuospatial tracking task resulted in large multitasking costs (main effect of single-task vs. multitask: mean multitasking cost = 15.58%; *F*(1,37) = 202.77, *p* ≤ .001, *η* ^2^_P_ = .846). Importantly, as seen in Table 2, there were no multitasking cost baseline differences between the two training groups (*t*(37) = −0.11, *p* = .91).

**Table 1:** Summary data for the training tasks collapsed across groups

	Pre-training	Post-training	Pre-to-post		Bayesian ANOVA BF_10_
	*M*(SD)	*M* (SD)	*F*	*p*	
Overall dual-task accuracy cost	15.58 (7.81)	12.84 (6.83)	4.46	**.04**	1.25
Dual-tracking cost	−0.46 (4.57)	−0.10 (2.07)	0.24	.63	0.26
Dual discrimination accuracy cost	16.18 (7.10)	12.95 (6.44)	7.26	**.01**	2.58
Dual discrimination RT cost	12.83 (20.11)	16.45 (10.78)	1.23	.27	0.43
Single tracking accuracy (%)	80.20 (3.62)	85.76 (3.16)	67.61	**<.001**	1.54
Dual tracking accuracy (%)	80.65 (5.29)	85.86 (2.66)	37.63	**<.001**	448345.66
Single discrimination accuracy (%)	83.25 (6.36)	87.79 (6.60)	13.45	**.01**	57.86
Dual discrimination accuracy (%)	67.07 (8.29)	74.84 (8.38)	45.21	**<.001**	65672.91
Single discrimination RT (ms)	326.29 (32.12)	320.88 (28.02)	7.87	**.01**	5.63
Dual discrimination RT (ms)	339.13 (40.76)	337.34 (34.42)	0.28	.60	0.26

*Note*: The table shows the mean standard deviation (SD) and mean, and training-related changes for the overall dual-task cost (overall dual cost, %), dual tracking cost (%), dual discrimination cost (%), dual discrimination RT cost (ms), single tracking accuracy (%), dual tracking accuracy (%), single discrimination accuracy (%), dual discrimination accuracy (%), single discrimination RT (ms), and dual discrimination RT (ms). Bold text indicates significant effect at *p* < .05 level

**Table 2:** Summary data for the training tasks by group

	Dual-task group		Single-task group		Group by session					Bayesian ANOVA BF_10_
	Pre-training	Post-training	Pre-training	Post-training	Baseline group comparisons					
	*M* (SD)	*M* (SD)	*M* (SD)	*M* (SD)	*t*	*p*	*F*	*p*	^*η2P*^	
*Overall dual cost*	15.86 (6.21)	8.79 (4.72)	15.58 (9.25)	16.69 (6.33)	−0.11	.91	8.42	**.01**	.185	0.88
Dual tracking cost	−1.48 (1.74)	−0.45 (1.65)	0.51 (6.08)	0.22 (2.41)	1.40	.18	0.77	.39	.020	23.55
*Dual discrim cost*	17.34 (6.23)	9.24 (4.49)	15.07 (7.84)	16.47 (6.09)	−1.00	.32	14.62	**<.001**	.283	0.09
Dual discrim RT cost	15.81 (21.2)	16.04 (9.68)	10.00 (19.09)	16.84 (11.9)	−0.90	.38	1.08	.31	.028	22.92
Single tracking accuracy (%)	80.70 (4.39)	85.93 (2.77)	79.72 (2.73)	85.60 (3.56)	−0.83	.41	2.35	.63	.006	33.67
Dual tracking accuracy (%)	82.18 (3.89)	86.37 (2.93)	79.21 (6.09)	85.38 (2.34)	−1.82	.08	1.36	.25	.036	17.97
Single discrim accuracy (%)	85.13 (6.51)	87.53 (6.54)	81.46 (5.82)	88.04 (6.82)	−1.86	.07	2.92	.10	.073	9.81
*Dual discrim accuracy*(%)	67.79 (9.50)	78.29 (7.74)	66.39 (7.13)	71.57 (7.79)	−0.52	.61	5.20	**.03**	.123	4.58
Single discrim RT (ms)	323.71 (29.4)	318.66 (26.88)	328.75 (35.05)	322.99 (29.6)	0.49	.63	0.03	.85	.001	32.06
Dual discrim RT (ms)	339.52 (39.30)	334.71 (31.80)	338.75 (44.05)	339.83 (37.4)	−0.06	.95	0.71	.41	.019	24.17

*Note*: The table shows the mean SD and mean overall dual-task cost (overall dual cost, %), dual tracking cost (%), dual discrimination cost (dual disrim cost, %), dual discrimination reaction time cost (dual discrim RT cost, ms), single tracking accuracy (%), dual tracking accuracy (%), single discrimination accuracy (single discrim accuracy, %), dual discrimination accuracy (dual discrim accuracy, %), single discrimination RT (single discrim RT, ms), and dual discrimination RT (dual discrim RT, ms). Bold text indicates significant effect at *p* < .05 level

**Fig. 1:**
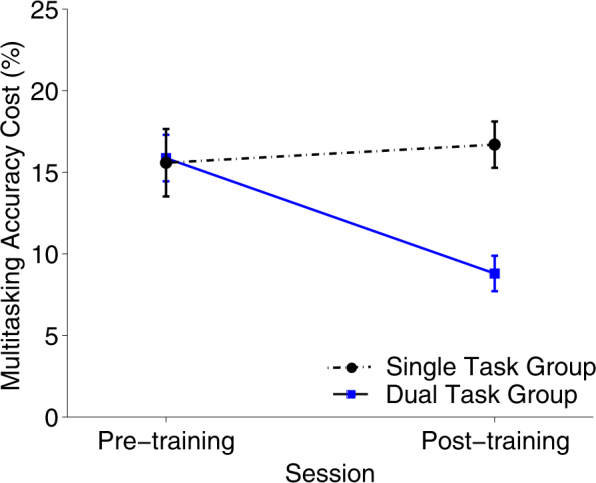
Multitasking costs (dual−sing tracking + dual−sing detection) pre-training and post-training as a function of group (multitasking, single task) showed a session × group interaction (*F*(1,37) = 8.42, *p* = .006, *η* ^2^_P_ = .185), indicating that gaining trains were specific to the multitasking training group. Multitasking cost error bars represent SEM

To determine the effect of multitask and single-task training on multitasking performance, a mixed-design session (pre-training, post-training) × group (multitasking, single) ANOVA revealed a main effect of session (*F*(1,37) = 4.46, *p* = .042, *η* ^2^_P_ = .108), indicating that multitasking performance improved significantly from pre-training to post-training. Critically, as seen in Fig. 1 and Supplementary Table 1, the improved multitasking performance was specific to the multitasking training group, as reflected in the significant session × group interaction (*F*(1,37) = 8.42, *p* = .006, *η* ^2^_P_ = .185), even though both groups showed significant pre-to-post-training improvements in their respective training-related tasks (Table 2). Bayesian ANOVAs revealed that a model in which the group interaction was added to the model explained the data better (BF_12_ = 0.88 in favour of the main effects model) than a main effects model (i.e., session and group entered separately). These results replicate previous findings,^2,3,7,9–11,42^ and demonstrate that multitasking training resulted in a significant decrease in multitasking cost relative to the control group, who trained on the two tasks in isolation.

### Training data

During the training phase, participants in the multitasking group performed the shape discrimination task and visuomotor tracking task concurrently (72 three-minute trials over six sessions), while the single-task training group spent their time equally on the single visuomotor tracking task (36 three-minute trials over six sessions) and shape discrimination task (36 three-minute trials over six sessions).

In order to assess if training was equally enjoyable for the multitasking and single-task group, participants rated their training experience on a scale from 1 (minimally) to 10 (maximally) at the end of the final session. Crucially, there was no significant difference between the multitasking group (*M* = 8.26) and the single-task group (*M* = 7.45; *t*(37) = 1.70, *p* = .10; BF_10_ = 0.96).

**Fig. 2:**
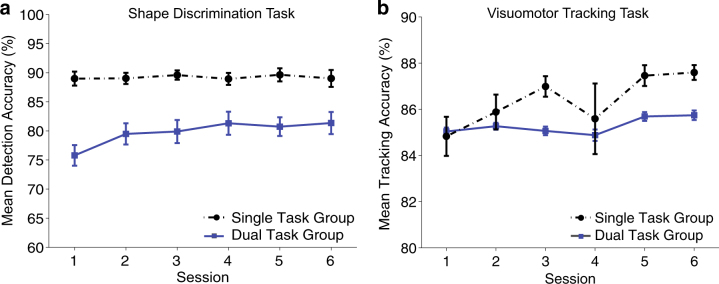
Training data by group (multitasking, single-task) across the six training sessions. **a** shows the mean visuomotor tracking accuracy and **b** shows the mean shape discrimination accuracy (%). Error bars represent SEM

To investigate whether there were any group differences in overall performance gains across the six training sessions, we conducted a series of mixed-design ANOVAs. A 6 (session) × 2 group (single vs. multitasking training) ANOVA for discrimination accuracy revealed a significant main effect of session (*F*(5,185) = 3.64, *p* = .004, *η* ^2^_P* = *_.090), indicating that overall performance improved across successive training sessions. Given the relatively easy nature of the task, the single-task training group reached asymptote after the first training session and performed the shape discrimination task at ceiling across the remaining sessions (Fig. 2; Supplementary Table 2). The resultant significant interaction between session × group revealed that the increase in accuracy was significantly higher for the multitasking training group (mean change = 5.56%) than for the single-task training group (mean change = 0.02%, *F*(5,185) = 3.24, *p* = .008, *η* ^2^_P* = *_.081). Bayesian ANOVAs confirmed this pattern of results and weakly favoured the model that included a session by group interaction (BF_12_ = 2.25 in favour of the main effects model).

Analyses of tracking accuracy revealed a main effect of session (*F*(5,185) = 2.59, *p* = .027, *η* ^2^_P_ = .065), such that overall tracking accuracy significantly increased from Session 1 (*M* = 85%) to Session 6 (*M* = 87%), and did not differ between groups (*F*(5,185) = .98, *p* = .434, *η* ^2^_P_ = .026). Bayesian ANOVAs revealed no evidence for group differences (BF_12_ = 89.58 in favour of the main effects model). The results therefore indicate similar visuomotor tracking performance across the six sessions for both training groups.

### Cognitive tasks

As shown in Table 3 and Supplementary Table 3, standard effects were observed in all six tasks, with no significant baseline differences between the two training groups (all *p*s > .21). To explore the benefits of multitask and single-task training on other action control measures, the sample was first assessed as a whole (Table 4 and Supplementary Table 3). Significant main effects of training on response selection and response inhibition tasks were only observed for the single response selection task and the Go-Nogo task (all *p*s < .01).

**Table 3:** Summary data for the transfer tasks by group

	Multitasking group		Single-task group							Bayesian ANOVA BF_12_
	Pre-training	Post-training	Pre-training	Post-training	Baseline group comparisons		Group by session			
	*M*(SD)	*M* (SD)	*M* (SD)	*M* (SD)	*t*	*p*	*F*	*p*	^*η2P*^	
*Action selection tasks*										
Single RS Task (RT)	643.39 (88.28)	586.74 (72.57)	619.73 (90.71)	582.41 (90.21)	−0.83	.41	1.62	.21	.042	16.99
AB mag	22.59 (15.03)	17.87 (14.95)	21.46 (13.25)	22.50 (13.71)	−0.25	.81	1.18	.29	.031	20.0
PRP mag	213.18 (97.87)	216.76 (127.47)	243.96 (213.40)	200.09 (209.52)	0.58	.56	2.15	.15	.055	13.76
Stroop effect	126.71 (93.35)	102.57 (76.02)	116.85 (76.95)	93.86 (91.03)	−0.36	.72	0.01	.97	.000	31.83
Flanker effect	86.29 (42.99)	81.13 (23.11)	96.75 (38.93)	94.12 (52.65)	0.80	.43	0.07	.80	.002	31.19
Nogo com. error (%)	23.25 (15.47)	26.02 (15.69)	17.22 (13.95)	25.41 (12.40)	−1.27	.21	1.73	.20	.045	16.59
Go-Nogo RT (ms)	312.14 (26.73)	298.66 (36.97)	322.69 (43.64)	298.82(32.92)	0.92	.37	1.30	.26	.034	18.76

*Note*: The table shows the mean SD and mean single response selection RTs (ms), baseline group comparisons and changes in task performance for each transfer task as a function of group (multitasking group and single-task group): Attentional Blink magnitude (AB mag), Psychological Refractory Period magnitude (PRP mag), Stroop effect (ms), Flanker effect (ms), Go-Nogo Commission (Nogo com.) error (%), and Go RTs (ms)

**Table 4:** Summary data for the transfer tasks collapsed across groups

	Pre-training	Post-training	Pre-to-post		Bayesian ANOVA BF_10_
	*M* (SD)	*M* (SD)	*F*	*p*	
*Action selection tasks*					
Single RS Task (RT)	631.26 (89.16)	584.52 (81.05)	38.18	**<.001**	23888.00
AB magnitude	22.01 (13.97)	20.25 (14.33)	0.48	.49	0.28
PRP magnitude	228.96 (165.98)	208.21 (172.39)	1.55	.22	0.46
Stroop effect	121.66 (84.34)	98.10 (83.07)	1.71	.10	0.78
Flanker effect	91.65 (40.76)	87.79 (41.02)	0.63	.43	0.30
Go-Nogo Go RT (ms)	317.55 (36.32)	298.74 (34.48)	16.78	**<.001**	107.76
Go-Nogo com. errors (%)	20.16 (14.83)	25.71 (13.91)	−7.09	**.01**	4.15

*Note*: The table shows the mean SD, mean, and training-related changes for all transfer tasks: single response selection (RS) RTs (ms), Attentional Blink (AB) magnitude, Psychological Refractory Period (PRP) magnitude, Stroop effect (ms), Flanker effect (ms), Go-Nogo Commission error (%), and Go RTs (ms). Bold text indicates significant effect at *p* < .05 level

To investigate the effect of group membership on transfer (Table 3 and Supplementary Table 3), mixed-measure ANOVAs examining session (pre-training, post-training) × group (single vs. multitasking training) showed no significant training-related differences between groups on all outcome measures (*p *> .15). Bayesian ANOVAs confirmed this pattern of effects for all outcome measures, such that main effects models, in which session and group were entered separately, were preferred to session × group models (BF_12_ ranging from 13.76 to 31.83 in favour of the main effects model). In summary, these analyses provide strong evidence that the training-related benefits generated from our protocol do not transfer to any of the other action control measures employed.

## Discussion

In this study, we investigated the effects of a dynamic dual-task training regime on multitasking performance and further assessed whether training-related benefits generalize to other untrained action control tasks that have previously been shown to share underlying or related mechanisms with the trained tasks.^47^ We had participants train on a concurrent perceptual discrimination and visuomotor tracking task (multitasking group), while the single-task group performed these two component tasks in isolation. We had two main goals in conducting this study. First, in line with previous dual-task findings,^2,3,7,9–11,42^ we aimed to examine whether extensive multitasking training in a stimulating and high-interference environment leads to training-related benefits. Multitasking performance before and after training was examined by quantifying the difference between the multitasking trials and single-task trials. Consistent with previous studies, our findings showed that multitasking performance selectively improved for participants that trained exclusively on the combined visuomotor tracking and perceptual discrimination task (multitasking group) and not for the active control group that trained on the two component tasks in isolation. Importantly, this training-related benefit was not due to initial performance differences between the two groups at baseline, as both groups showed similar costs when performing both tasks simultaneously. In addition, we also found training gains on the trained tasks irrespective of group condition. The results therefore provide support that training on a dynamic dual-task induces the learning of task-specific skills.

Second, by employing a wide array of cognitive tasks pre-training and post-training, we were able to show that the observed dual-task training effects from this combined dynamic visuomotor tracking and perceptual discrimination task did not generalize to our specific set of action control tasks. Specifically, while standard effects for each measure were observed pre-training and post-training, we did not find any evidence of transfer for dual-task training effects on the PRP or any other response selection measures. Given that all response selection transfer tasks included new visual stimuli and, as in the case of the PRP, a different input modality (auditory stimuli for the second task), the results suggest that training leads to learning on how to coordinate stimulus and modality-specific information more efficiently. The results are in line with previous dual-task^8,28^ and action game studies^37,42^ that showed no significant evidence of transfer to other dual-task conditions. There was also no evidence of training-related enhancements to tests of response inhibition (Go-Nogo) and spatial attention (Flanker). An overall small decrease in Go RTs on the Go-Nogo task was observed for both groups post-training, but the accompanying overall increase in commission errors indicates that this reduction was likely driven by a speed/accuracy trade-off.

Overall, the pattern of results is largely consistent with the dual-task training^8,18,29–31^ and brain training literature (for a review, see ref. 48), which finds little evidence that multitasking training generalizes beyond task-specific skills and further contradicts previous findings in the video game training literature that found training-related enhancements of executive control skills.^34,38,42^ The absence of transfer on learning seems inconsistent with the neuronal overlap theory, which postulates that transfer may occur if the trained tasks and transfer tasks draw on common neural substrates.^24–26^ When considering executive control of actions, a reasonable prediction of this theory is that if two different action control tasks draw on the same frontoparietal and subcortical system, then improving the process of the trained tasks that taps this brain region should also result in improved effects for other action control processes subserved by that system. In contrast, we found that training on two tasks simultaneously reduced the dual-task costs on the exact trained tasks but this training benefit did not generalize to other action control tasks. Thus, the absence of a training-induced effect on any transfer task and the presence of a task-specific multitasking training effect is in keeping with the idea that repeated exposure to particular tasks or stimuli may facilitate more efficient resource allocation, and in turn more optimal dual-task performance in the practiced situation.

As multitasking costs only improved when the two tasks were practiced in combination, our data imply that the critical aspect of decreased dual-task cost may lie in allowing the brain to learn how two specific tasks can be coordinated efficiently.^49^ However, future research is needed to develop a greater understanding of the underlying mechanisms that drive training-related neural plasticity.

To date, relatively little is known about the underlying mechanisms that give rise to positive transfer effects. While our current intervention employed a dynamic dual task to tax action selection processes in a context that is often found in real-world situations, it differs significantly from interactive video action games that require participants to intentionally vary their task priorities among many dimensions in order to achieve the required goals and sub-goals. To successfully perform these games, participants are inherently forced to form judgments about the underlying rules and relationships that exist between the different tasks, which has recently been shown to lead to transfer to new stimulus sets.^28^ This suggests that the brain may use the abstract association between tasks to transfer learning at the response selection/decision-making stage. Further work is needed to clarify whether training regimes that require the formations of abstract rules leads to positive transfer in other executive domains.

In sum, by using a rigorous intervention design and dynamic, continuous multitasking paradigm game, we were able to demonstrate that repeated exposure to two combined tasks induces the learning of task-specific strategies that do not generalize to a wide range of action control tasks. Instead, our findings indicate that a training-related decrease in dual-task interference may be dependent on learning concrete stimulus-response associations so that two specific tasks can be coordinated more effectively,^49^ thus allowing response selection to become more automatized with training and less prone to the negative effects that occur when central resources are taxed by concurrent load.^50^

## Methods

Participants attended ten sessions, one per day with two rest days included. The first, second, ninth, and tenth sessions were the pre-training and post-training sessions, while the remaining six sessions were training session. Performance was compared pre-training and post-training between a multitasking training group who had trained on a perceptual discrimination task while performing a simultaneous visuomotor tracking task, and an active control group (single-task training group) who had completed the component tasks in isolation. Methods were performed in accordance with relevant regulations and guidelines.

### Ethics statement

Informed consent from each participant was obtained after a full explanation of the experiment, according to the procedures approved by the University of Queensland’s Research Ethics Committee.

### Participants

Minimum sample sizes were calculated to achieve 80% power (*f *= .3) to detect a significant 2 (training group) × 2 (session) interaction if a true effect was present. Power calculations revealed that a minimum sample size of 14 participants per group would be required. We based our combined sample size on this calculation and the sample size of our previous training studies.^9,28^

Data were recorded from 47 adults aged between 18 and 40 years (40 females, mean age = 22). All participants were right-handed, had normal or corrected to normal vision, with no history of neurological, vascular or psychiatric disorder, and were not taking any hypertension or psychotropic medications. All participants were recruited through The University of Queensland’s paid research participation pool and received AUD10 per hour for participation and were further able to earn bonus dollars for performance. The analyses reported here stem from 39 participants as one participant was excluded for not completing all ten sessions of the study and an additional seven participants were excluded for poor performance (more than 3 SDs above the RT or below the accuracy mean) in one or more of the six cognitive tasks during pre-training. These exclusion procedures were determined a priori.

### Materials and apparatus

The experiment was run on an Apple Mac Mini running Matlab (The MathWorks, Natick, MA) and the Psychophysics toolbox extension^51^ was employed to control the display of stimuli on a 21” CRT monitor (100 Hz refresh rate) and for data collection. For all the tasks, responses were registered through a standard Apple USB keyboard^52^ and participants viewed the monitor from an approximate distance of 57 cm.

### Procedure

#### Pre-training sessions

**Fig. 3:**
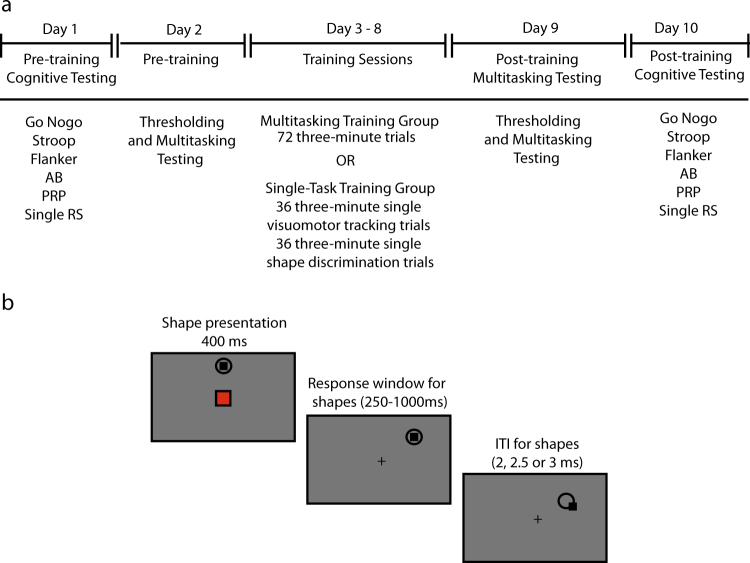
Experiment design overview. **a** Outline of the experimental paradigm. Participants first took part in a pre-training phase in which they completed a battery of cognitive tasks (Day 1), thresholding and multitasking testing (Day 2). Participants then trained on either a combined visuomotor tracking and shape discrimination task (multitasking group) or on both component tasks in isolation (single-task group) over 6 days. Two post-training sessions were conducted, in which participants again undertook the multitasking tasks (Day 9) and the battery of cognitive tasks (Day 10). **b** Trial outline for the visuospatial tracking and shape discrimination task

The paradigm was divided into three phases as illustrated in Fig. 3. Participants were randomized to either the multitasking training group that trained on a continuous visuomotor tracking and an intermittent shape discrimination task simultaneously (*N* = 19) or an active control group (single-task training group) that trained on both component tasks in isolation (*N* = 20). A no-contact control group was not included, as these groups often differ significantly in terms of motivation and expectancy effects.^44^ Importantly, age (*t*(37) = .35, *p* = .73) and gender break down (*t*(37) = .41, *p* = .67) did not differ between the groups. In order to avoid expectancy differences across the groups, we told each participant that the training paradigm was created to investigate the effects that training has on cognitive control functions.

In phase 1, participants completed two pre-training sessions, administered one day apart from each other, lasting approximately 1.5–2 h each. On day 1, participants were administered a battery of seven tasks^47^ in order to test the cognitive impact of training to other closely related action control processes. An index of multitasking was provided by the PRP, whereas other response selection, inhibitory control, and selective attention measures were provided by the Stroop task, six AFC single response selection task, AB task, Go-Nogo, and Flanker task (please refer to the Transfer Task section for a more comprehensive description of each task). Participants also completed a stop-signal task. However, these data were discarded due to a technical issue. All tasks were completed in randomized order.

In the second pre-training session, participants first took part in an adaptive staircase procedure to determine the individual difficulty levels in the visuomotor tracking (nine 60 s trials) and shape discrimination task (nine 120 s trials), so that each participant performed the two tasks at ~80% accuracy.^42^ These difficulty levels were then utilized to establish the parameters of the two tasks in the multitasking condition, so that each participant performed the multitask condition at their own individual difficulty level. Following baseline thresholding evaluation, participants ran through five 3-min trials of each condition (i.e., single shape discrimination task condition, single visuomotor tracking task condition, and a concurrent shape discrimination and visuomotor tracking multitask condition) to assess multitasking costs at baseline. At the end of each trial, the overall percentage of time spent within the moving target disc (single visuomotor tracking task and dual-task condition) and the mean RT and proportion of correct responses to all shapes (single detection and dual-task condition) were displayed.

#### Training sessions

In phase 2, participants trained for 3 consecutive days, followed by 2 days of rest and another 3 consecutive days of training. Each session included twelve 3-min trials (72 trials in total across the six sessions), in which participants in the multitasking group exclusively performed the visuomotor tracking and shape discrimination task concurrently, while participants in the single-task training group divided their time equally between the single shape discrimination task (6 × 3-min trials) and the single viusomotor tracking task (6 × 3-min trials), with task order randomized across participants. After each trial, participants in both training groups received performance feedback. The performance feedback in the multitasking group consisted of the participants’ mean visuomotor tracking accuracy and shape discrimination accuracy, whereas performance feedback in the single-task group was trial-type dependent. To maximize motivation and in line with previous training paradigms,^2,9^ participants in both groups were awarded bonus points. To encourage equal task engagement of each component task, participants in the multitasking training group received one bonus point if performance on both component tasks was over 70%, while participants in the single-task training group received one bonus point if performance was over 85% in the single visuomotor tracking or single shape discrimination accuracy. In contrast, if accuracy was below the designated values, participants in both groups lost a bonus point.

#### Post-training phase

The third phase started one day after completion of training. On day 1, participants ran through both single-tasking and multitasking trials (same procedure as the testing phase in phase 1, session 2) to assess the impact of single-task or multitask training on multitasking performance. On the final day, participants completed the same seven cognitive tasks as on day 1 (phase 1) in order to measure transfer to other action control tasks. All ten sessions took place at approximately the same time of day for each participant.

### Tasks and stimuli

#### Trained tasks

We followed the general techniques of Anguera and colleagues^42^ to assess multitasking performance in a dual visuomotor tracking and shape discrimination task. To ensure equal visual stimulation between the multitasking and single-task conditions, shapes were presented in the center of the screen in the single visuomotor tracking task but participants were instructed to ignore these discrimination stimuli. Similarly, a moving target disc was presented during the shape discrimination task, with participants asked to ignore the moving disc.

#### Visuomotor tracking task

During each tracking epoch, participants had to continuously pursue a visually presented moving black target disk (0.50°) with the mouse so that the black cursor (0.10°) remained as close as possible to the center of the moving target, while ignoring the presentation of shapes in the center of the screen (see Fig. 3). At trial onset the cursor was positioned within the center of the target disc, which was located at the top center of the screen. The participants’ right hand held the mouse through which they could control the motions of the cursor and the left hand was used to initiate the start of each trial by pressing the spacebar on the keyboard. The trajectory of the target was generated by the visible target bouncing off the edge of the display or off two invisible discs in a Newtonian direction. Movements in the *x*-axis and *y*-axis were calculated independently at a screen resolution of 1024 by 768 pixels. A 1 cm protective radius ensured that the tracking target did not overlap with the centrally presented shapes.

#### Visual shape discrimination task

Each trial began with an instruction screen that displayed the coloured target shape for the upcoming trial and further reminded participants to respond as quickly as possible by pressing the space bar on a keyboard when the target stimulus matched the one presented on the screen, while ignoring the appearance of distractor shapes. For each trial, a new target was randomly drawn from 12 possible coloured (red, green, blue, or yellow) shapes (square, hexagon, or star), with the remaining non-target shapes serving as distractors. Target presentation occurred at 50% probability with shapes randomly presented for 400 ms in the center of the screen every 2, 2.5, or 3 s.

#### Dual task

In the dual-task condition, participants were required to perform the shape discrimination task and visuomotor tracking task simultaneously.

### Thresholding procedure

To ascertain that multitasking deficits are not due to individual differences in task skills, participants first underwent an adaptive thresholding procedure (pre-training phase, session 2) to determine an individual difficulty level that would result in ~80 accuracy in each single-task condition. To determine the best RT window and tracking speed to keep accuracy at ~80%, a standard regression technique over the visuomotor tracking and discrimination thresholding trials was calculated separately at the end of the thresholding blocks and the resultant levels were then utilized for the remainder of the experiment. Specifically, we employed standard regression techniques (ordinary least squares) to determine the slope and intercept between the thresholding step level and accuracy [slope, intercept] = regression (trial accuracy, thresholding step). Once the linear function was identified, the thresholding step that corresponds to the desired 80% accuracy was calculated [test threshold step] = round (slope × median accuracy score + intercept).

During both visuomotor tracking and shape discrimination thresholding, the difficulty of the task changed if accuracy was above 82.5% or below 77.5% at the end of the trial. In order to achieve ~80% accuracy, an adaptive algorithm calculated proportional level changes that were dependent on how close participants performed above or below the 80% criterion, with each 1.75% increment away from the criterion resulting in a level change. Both staircases comprises 49 levels.

All participants started with 9 × 60 s visuomotor tracking trials. For the visuomotor tracking task the different levels represented the minimum (.0100 dps, level 1) and maximum (.0112, level 49) speeds at which the target was moving across the screen. Changes in target speed levels corresponded to 0.0040 increments if the target speed was between 0.0100 (level 1) and 0.0820 (level 19), whereas 0.0010 increments were employed for target speeds between 0.0820 (level 19) and 0.1110 (level 49), with all participants starting with an initial tracking speed of 0.920 (level 29). Thus, if a tracking speed of 0.920 resulted in a visuomotor tracking accuracy of 86%, then tracking speed would increase by .002 dps ((86−80%)/1.75) in the subsequent trial, whereas a 74% performance would result in a .003 decrease in target speed. Continuous performance feedback was provided in the thresholding phase only and consisted of the cursor turning red as soon as the cursor moved outside the radius of the moving target disk.

The visuomotor tracking threshold procedure was followed by 9 × 20 s trials of the visual shape discrimination task. For the shape discrimination task, the same staircase algorithm was employed to determine the RT window in which the proportion of correct responses to all shapes (i.e., correctly responding to the target shape and correctly inhibiting a response to all distractor shapes) converged to the ~80% criterion. Each level signified the total amount of time that a participant had to respond to the presented target shape. The RT windows ranged from 1000 ms (level 1) to 250 ms (level 49) and was initially set at 450 ms RT window (level 29). If performance on a given trial was greater than the criterion, a shorter RT window (shortest window = 250 ms, level 49) was calculated, whereas performance below the criterion led to a longer RT window (longest window = 1000 ms) for the subsequent trial. Specifically, RT window changes between 1000–550 ms resulted in 25 ms level changes, whereas changes in RT windows between 550–250 ms corresponded to 10 ms level changes.

A centrally presented fixation square provided performance feedback during the thresholding session. The fixation square turned green for 50 ms if participants responded correctly to the target shape within the allocated response-window or when a response to a non-target distractor shape was successfully suppressed (see thresholding procedure). In contrast, if participants failed to respond to the target shape within the allocated response-window or responded incorrectly to a non-target shape, the fixation square would turn red for 50 ms.

### Transfer tasks

#### Psychological refractory period

In this task, participants first learned the stimulus-response mappings of two different sensorimotor tasks (20 trials per block). In the first training block, participants learned the stimulus response mappings of a visual discrimination task (Task 1), which required participants to respond to one of four different symbols (%, @, &, or #) via key press. In the second training block, participants followed the same task structure as in Task 1, except that the visual discrimination task changed to an auditory discrimination task (Task 2). Here, participants responded via key press to one of four different complex tones. The final practice block was identical to the trials in the experimental phase. Each trial began with the presentation of a Task 1 (T1) symbol at the center of the screen and after either a short (200 ms) or long (1000 ms) stimulus onset asynchrony (SOA) the second task (T2) auditory target was presented. Stimuli remained on-screen for 200 ms and participants were asked to respond as fast as they could to the two tasks while maintaining accuracy and without grouping their responses. Next, participants started the experimental phase, which consisted of four testing blocks, each containing 40 trials equally divided between the two SOA conditions. The dependent measure was the PRP effect. The effect was computed by subtracting the obtained RT of the second task in the 1000 ms condition from the RT in the 200 ms condition. Better performance was indicated by lower differences scores.

#### Single response selection task

In this task participants were required to discriminate between six different coloured fractals via key press. Each stimulus was mapped to a specific response key and hand (A, S, or D for left-hand responses and J, K, or L for right-hand responses), with the mapping of hand to task counterbalanced across participants. In each trial, the central fixation cross was followed by one of the fractal stimuli for 200 ms. Participants were instructed to respond as quickly and as accurately as possible via key press to the stimulus. Prior to the experimental phase, participants first completed 36 practice trials and then moved on to the experimental phase (four blocks of 36 trials). The key dependent variable was the RT, with lower RTs indicating better performance.

#### Stroop task

In this classic Stroop task, participants were instructed to report the corresponding ink color of four coloured (“blue”, “red”, “yellow”, “green”) and four non-coloured (“saucer”, “fork”, “cup”, “spoon”) words via key press. Each trial began with the presentation of the fixation cross, which was replaced by the presentation of a word target (500 ms). Three different types of trials were conducted: (1) in congruent trials the printed color word matched the ink color (e.g., “red” printed in red); (2) in incongruent trials the printed color word and ink color mismatched (e.g., “red” printed in blue ink color); and (3) in neutral trials a non-color word was printed in any of the four colors (e.g., “cup” printed in yellow ink color). The word target was equally likely to either be congruent, incongruent, or neutral, with the order of trial types randomized. Prior to the experimental phase (four blocks of 36 trials), participants completed 24 response-mapping practice trials. The key dependent measure was the “Stroop congruency effect”. This effect was calculated by computing the mean RT difference between congruent and incongruent trials. Lower difference scores represent better performance.

#### Attentional blink

Each trial consisted of a rapid serial visual presentation (RSVP) stream comprising of two targets (letters of the alphabet, excluding I, L, O, Q, U, V, X) and eight digits (ranging from 2 to 9) serving as distractors. Participants were instructed to report the identity of the two target letters at the conclusion of the RSVP stream. There was no speeded response pressure and participants were told to guess if unsure. Each stimulus was presented for 100 ms. Target 1 (T1) was presented at serial position 3 with Target 2 (T2) following T1 after either 200 ms (lag 2), 300 ms (lag 3), 500 ms (lag 5), or 700 ms (lag 7). After 24 initial practice trials, participants performed four test blocks (24 trials per block). To calculate the AB magnitude (dependent measure), we subtracted the mean of lags 2 and 3 T2|T1 accuracy from the mean of lags 5 and 7. A smaller magnitude of the AB deficit represents better performance.

#### Go-Nogo task

In the Go-Nogo task, participants were instructed to discriminate between two white, abstract 3D shapes. One shape represented the go stimulus and the other shape represented the no-go stimulus. The participants’ task was to press the “G” key on a computer keyboard as soon as the go stimulus appeared in the center of the screen, but to withhold the response if the no-go stimulus appeared (25% of trials). Go and no-go stimuli were counterbalanced across participants with speed and accuracy equally emphasized. Each trial began with a fixation cross (200 ms), followed by one of the two stimuli (200 ms) and an 1800 ms response window. After completing 24 familiarization trials, participants completed four test blocks (36 trials per block). The key dependent measure of interest was the proportion of commission errors on no-go trials (i.e., failure to stop a response). Fewer commission errors indicated better response inhibition.

#### Flanker task

The participants’ task was to indicate in which direction a central arrow target (> or <), flanked on both sides by two arrows, was pointing. Participants made their responses by pressing the “>” key and the “<” key for rightward-pointing arrows targets and leftward-pointing arrows, respectively. On congruent trials, the flankers were two arrows pointing in the same direction as the target arrow on each side (e.g., >>>>>). On incongruent trials, the flankers were arrows pointing in the opposite direction as the target arrow (e.g., >><>>). Finally, on neutral trials the target arrow was flanked by horizontal lines (e.g., --<--). After the presentation of a fixation cross, the five-arrow array appeared and remained on screen for 200 ms. Participants received 24 practice trials, followed by four experimental blocks (36 trials per block). There were an equal number of trials per condition and the order of trial types was randomly intermixed. The dependent measure was the “flanker congruency effect”. This effect was computed by calculating the mean RT difference between the congruent and incongruent trials. Lower RT difference scores indicated better performance.

### Data analysis

In order to evaluate the strength to which the evidence points toward or against the null hypothesis, we employed Bayesian methods in addition to traditional null hypothesis significance testing.^53^ This was important for the present study since it was likely that at least some of the tasks would show no effect of training. Thus, we needed to be able to assess evidence favoring the null.

#### Cognitive tasks

To establish whether the observed performance during the pre-training session showed the standard effects for the Go-Nogo (Go RTs, Nogo commission errors), Stroop (Stroop Congruency Effect), Flanker (Flanker Congruency Effect), Single response selection (RS), PRP (PRP effect), and AB (AB magnitude) tasks, we first evaluated performance (accuracy and/or RTs) using mixed ANOVAs with the relevant key measures of interest at baseline (Session 1) as a within-subject factor and group (single, multitasking) as between-subject factor. Training-related transfer effects were quantified as a reduction in the standard effects between pre-training and post-training sessions.

In order to quantify the degree to which the evidence supports the null hypothesis (i.e., training-related improvements transfer to other action control tasks) or the alternative (i.e., training-related improvements do not transfer to other action control tasks), Bayesian mixed-measures ANOVAs were conducted in JASP^54^ for each transfer task. Inverse Bayes Factors (BF_10_) were used to denote that prior odds should be updated by a factor of 10 in favour of the alternative hypothesis (multitasking training has an effect) compared to the null (multitasking training has no effect). To compare the posterior probability of a null model (a model without a session by group interaction) against a model including the interaction, we used the fact that Bayes factors are transitive. Thus, by transitivity we divided the model with main effects (BF_10_) by the model that adds the group interaction (BF_20_), with the resultant BF_12_ indicating the evidence in favour of the main effects model. Overall, BF of 1–3 signifies weak evidence, BF 3–10 indicates substantial support, and BF > 10 correspond to strong evidence.^55^

#### Multitasking performance

Multitasking performance was estimated as the difference between the multitasking trials and single-task trials [(Dual-task detection trials—single-task detection trials) + (dual-task tracking trials—single-task tracking trials)], with smaller scores indicating better multitasking performance (i.e., less interference when engaging in the two tasks simultaneously). To examine differences at baseline, performance (multitasking cost, perceptual discrimination performance, and tracking performance) across the two groups (multitasking, single) was examined using independent *t*-tests. Multitasking cost differences between groups were evaluated with a mixed design ANOVA (NHST and Bayesian) with session (pre, post) as within-subject factor and group (single, dual) as between-subject factor.

#### Training data

Mixed-measures 6 (session) × 2 group (single, multitasking) ANOVAs (traditional NHST and Bayesian) were conducted for each training task (perceptual discrimination accuracy and visuomotor tracking accuracy) to investigate whether there were any group differences in overall performance gains.

### Data availability

All training data are available as Supplementary Information, or upon reasonable request from the corresponding authors.

### Code availability

Code used throughout this study are available upon reasonable request from the corresponding authors.

## Electronic supplementary material

Supplementary Table Legends

Supplementary Table 1

Supplementary Table 2

Supplementary Table 3

## Electronic supplementary material

**Supplementary information** accompanies the paper on the *npj Science of Learning* website (10.1038/s41539-017-0015-4).

## References

[1] Dux PE, Ivanoff J, Asplund CL, Marois R (2006). Isolation of a central bottleneck of information processing with time-resolved FMRI. Neuron.

[2] Dux PE (2009). Training improves multitasking performance by increasing the speed of information processing in human prefrontal cortex. Neuron.

[3] Schumacher EH (2001). Virtually perfect time sharing in dual-task performance: uncorking the central cognitive bottleneck. Psychol. Sci..

[4] Sigman M, Dehaene S (2008). Brain mechanisms of serial and parallel processing during dual-task performance. J. Neurosci..

[5] Pashler H (1994). Dual-task interference in simple tasks: data and theory. Psychol. Bull..

[6] Welford AT (1952). The ‘psychological refractory period’ and the timing of high-speed performance—a review and a theory. Br. J. Psychol..

[7] Hazeltine E, Teague D, Ivry RB (2002). Simultaneous dual-task performance reveals parallel response selection after practice. J. Exp. Psychol. Hum. Percept. Perform..

[8] Liepelt R, Strobach T, Frensch P, Schubert T (2011). Improved intertask coordination after extensive dual-task practice. Q. J. Exp. Psychol..

[9] Garner KG, Tombu MN, Dux PE (2014). The influence of training on the attentional blink and psychological refractory period. Atten. Percept. Psychophys..

[10] Strobach T, Frensch PA, Soutschek A, Schubert T (2012). Investigation on the improvement and transfer of dual-task coordination skills. Psychol. Res..

[11] Van Selst M, Ruthruff E, Johnston JC (1999). Can practice eliminate the psychological refractory period effect?. J. Exp. Psychol. Hum. Percept. Perform..

[12] Garner KG, Dux PE (2015). Training conquers multitasking costs by dividing task representations in the frontoparietal-subcortical system. Proc. Natl. Acad. Sci. USA.

[13] Erickson KI (2007). Training-induced plasticity in older adults: effects of training on hemispheric asymmetry. Neurobiol. Aging.

[14] Miller EK, Cohen JD (2001). An integrative theory of prefrontal cortex function. Annu. Rev. Neurosci..

[15] Duncan J (2010). The multiple-demand (MD) system of the primate brain: mental programs for intelligent behaviour. Trends Cogn. Sci..

[16] Aron AR (2007). Converging evidence for a fronto-basal-ganglia network for inhibitory control of action and cognition. J. Neurosci..

[17] Aron AR, Poldrack RA (2006). Cortical and subcortical contributions to stop signal response inhibition: role of the subthalamic nucleus. J. Neurosci..

[18] Ruthruff E, Van Selst M, Johnston JC, Remington R (2006). How does practice reduce dual-task interference: integration, automatization, or just stage-shortening?. Psychol. Res..

[19] Pashler H, Baylis G (1991). Procedural learning: 2. Intertrial repetition effects in speeded-choice tasks. J. Exp. Psychol. Learn. Mem. Cogn..

[20] Logan, G. D. Toward an instance theory of automatization. *Psychol. Rev.* **95**, 492–527 (1988).

[21] Palmeri TJ (1999). Theories of automaticity and the power law of practice. J. Exp. Psychol. Learn. Mem. Cogn..

[22] Strobach T, Salminen T, Karbach J, Schubert T (2014). Practice-related optimization and transfer of executive functions: a general review and a specific realization of their mechanisms in dual tasks. Psychol. Res..

[23] Hirst W, Spelke ES, Reaves CC, Caharack G, Neisser U (1980). Dividing attention without alternation or automaticity. J. Exp. Psychol. Gen..

[24] Kuwajima M, Sawaguchi T (2010). Similar prefrontal cortical activities between general fluid intelligence and visuospatial working memory tasks in preschool children as revealed by optical topography. Exp. Brain Res..

[25] Lustig C, Shah P, Seidler R, Reuter-Lorenz PA (2009). Aging, training, and the brain: a review and future directions. Neuropsychol. Rev..

[26] Thorell LB, Lindqvist S, Bergman Nutley S, Bohlin G, Klingberg T (2009). Training and transfer effects of executive functions in preschool children. Dev. Sci..

[27] Lussier M, Gagnon C, Bherer L (2012). An investigation of response and stimulus modality transfer effects after dual-task training in younger and older. Front. Hum. Neurosci..

[28] Garner KG, Lynch CR, Dux PE (2016). Transfer of training benefits requires rules we cannot see (or hear). J. Exp. Psychol. Hum. Percept. Perform..

[29] Garner KG, Matthews N, Remington RW, Dux PE (2015). Transfer ability of training benefits differs across neural events: evidence from ERPs. J. Cogn. Neurosci..

[30] Strobach T, Liepelt R, Pashler H, Frensch PA, Schubert T (2013). Effects of extensive dual-task practice on processing stages in simultaneous choice tasks. Atten. Percept. Psychophys..

[31] Owen AM (2010). Putting brain training to the test. Nature.

[32] Strobach T, Frensch P, Muller H, Schubert T (2015). Evidence for the acquisition of dual-task coordination skills in older adults. Acta Psychol..

[33] Boot WR, Kramer AF, Simons DJ, Fabiani M, Gratton G (2008). The effects of video game playing on attention, memory, and executive control. Acta Psychol..

[34] Strobach T, Frensch PA, Schubert T (2012). Video game practice optimizes executive control skills in dual-task and task switching situations. Acta Psychol..

[35] Green CS, Sugarman MA, Medford K, Klobusicky E, Daphne B (2012). The effect of action video game experience on task-switching. Comput. Hum. Behav..

[36] Chiappe D, Conger M, Liao J, Caldwell JL, Vu KP (2013). Improving multi-tasking ability through action videogames. Appl. Ergon..

[37] Gaspar JG (2014). Are gamers better crossers? An examination of action video game experience and dual task effects in a simulated street crossing task. Hum. Factors.

[38] Green CS, Bavelier D (2003). Action video game modifies visual selective attention. Nature.

[39] Green CS, Bavelier D (2006). Enumeration versus multiple object tracking: the case of action video game players. Cognition.

[40] Wu S (2012). Playing a first-person shooter video game induces neuroplastic change. J. Cogn. Neurosci..

[41] Oei AC, Patterson MD (2013). Enhancing cognition with video games: a multiple game training study. PLoS ONE.

[42] Anguera JA (2013). Video game training enhances cognitive control in older adults. Nature.

[43] Engle RW, Tuholski SW, Laughlin JE, Conway AR (1999). Working memory, short-term memory, and general fluid intelligence: a latent-variable approach. J. Exp. Psychol. Gen..

[44] Shipstead Z, Redick TS, Engle RW (2012). Is working memory training effective?. Psychol. Bull..

[45] Mishra J, Anguera JA, Gazzaley A (2016). Video games for neuro-cognitive optimization. Neuron.

[46] Noack H, Lovden M, Schmiedek F (2014). On the validity and generality of transfer effects in cognitive training research. Psychol. Res..

[47] Bender AD, Filmer HL, Garner KG, Naughtin CK, Dux PE (2016). On the relationship between response selection and response inhibition: an individual differences approach. Atten. Percept. Psychophys..

[48] Simons DJ (2016). Do “Brain-Training” programs work?. Psychol. Sci..

[49] Schubert T, Szameitat AJ (2003). Functional neuroanatomy of interference in overlapping dual tasks: an fMRI study. Brain Res. Cogn. Brain Res..

[50] Logan GD (1978). Attention in character-classification tasks: evidence for the automaticity of component stages. J. Exp. Psychol. Gen..

[51] Brainard DH (1997). The psychophysics toolbox. Spat. Vis..

[52] Neath I, Earle A, Hallett D, Surprenant AM (2011). Response time accuracy in Apple Macintosh computers. Behav. Res. Methods.

[53] Rouder JN, Morey RD, Verhagen J, Swagman AR, Wagenmakers EJ (2016). Bayesian analysis of factorial designs. Psychol. Methods.

[54] Love, J. et al. JASP (Version 0.7) [Computer software] (2015).

[55] Kass, R. E., & Raftery, A. E. Bayes factors. *J. Am. Stat. Assoc*. **90**, 773–795 (1995).

