# Supplementary material for: Dynamic, continuous multitasking training leads to task-specific improvements but does not transfer across action selection tasks
Source: NPJ Sci Learn. 2017 Dec 4;2:14. doi: 10.1038/s41539-017-0015-4 (PMC6220332; doi:10.1038/s41539-017-0015-4)
Supplement: Supplementary file 1 — Supplementary Table Legends [file 41539_2017_15_MOESM1_ESM.docx]

**SUPPLEMENTARY TABLES**

**Supplementary Table 1**

This file contains the single vs. multitasking test data (Session 1 and 2). The data is related to Figure 2 and Table 1 and 3.

**Supplementary Table 2**

This file contains the training data across the six training sessions. The data is related to Figure 3.

**Supplementary Table 3**

This file contains the pre- and post-training data for the cognitive tasks (Stroop (Stroop_Task), Flanker (Flanker_Task), Go NoGo (Go_NoGo_Task, Pscyhological Refractory Period (PRP_Task), Single Response Selection (Single_6AFC_Task), Attentional Blink (AB_Task). The data is related to Table 3 and 4.
